# Protein deficiency reduces efficacy of oral attenuated human rotavirus vaccine in a human infant fecal microbiota transplanted gnotobiotic pig model

**DOI:** 10.1016/j.vaccine.2018.09.008

**Published:** 2018-10-08

**Authors:** Ayako Miyazaki, Sukumar Kandasamy, Husheem Michael, Stephanie N. Langel, Francine C. Paim, Juliet Chepngeno, Moyasar A. Alhamo, David D. Fischer, Huang-Chi Huang, Vishal Srivastava, Dipak Kathayat, Loic Deblais, Gireesh Rajashekara, Linda J. Saif, Anastasia N. Vlasova

**Affiliations:** a Food Animal Health Research Program, Department of Veterinary Preventive Medicine, Ohio Agricultural Research and Development Center, The Ohio State University, Wooster, OH 44691, USA; b Division of Viral Disease and Epidemiology, National Institute of Animal Health, National Agriculture and Food Research Organization, Tsukuba, Ibaraki 305-0856, Japan; c Division of Integrated Biomedical Sciences, School of Dentistry, University of Detroit Mercy, Detroit, MI, USA^1^

**Keywords:** Rotavirus, Vaccine, Malnutrition, Innate immunity, T cell, Microbiota

## Abstract

**Background:**

Low efficacy of rotavirus (RV) vaccines in developing African and Asian countries, where malnutrition is prevalent, remains a major concern and a challenge for global health.

**Methods:**

To understand the effects of protein malnutrition on RV vaccine efficacy, we elucidated the innate, T cell and cytokine immune responses to attenuated human RV (AttHRV) vaccine and virulent human RV (VirHRV) challenge in germ-free (GF) pigs or human infant fecal microbiota (HIFM) transplanted gnotobiotic (Gn) pigs fed protein-deficient or -sufficient bovine milk diets. We also analyzed serum levels of tryptophan (TRP), a predictor of malnutrition, and kynurenine (KYN).

**Results:**

Protein-deficient pigs vaccinated with oral AttHRV vaccine had lower protection rates against diarrhea post-VirHRV challenge and significantly increased fecal virus shedding titers (HIFM transplanted but not GF pigs) compared with their protein-sufficient counterparts. Reduced vaccine efficacy in protein-deficient pigs coincided with altered serum IFN-α, TNF-α, IL-12 and IFN-γ responses to oral AttHRV vaccine and the suppression of multiple innate immune parameters and HRV-specific IFN-γ producing T cells post-challenge. In protein-deficient HIFM transplanted pigs, decreased serum KYN, but not TRP levels were observed throughout the experiment, suggesting an association between the altered TRP metabolism and immune responses.

**Conclusion:**

Collectively, our findings confirm the negative effects of protein deficiency, which were exacerbated in the HIFM transplanted pigs, on innate, T cell and cytokine immune responses to HRV and on vaccine efficacy, as well as on TRP-KYN metabolism.

## Highlights

• Protein deficiency impacted immunity and reduced human RV vaccine efficacy.
• Human infant fecal microbiota exacerbated the negative effects of protein deficiency.
• Immunological dysfunction could have been induced by altered tryptophan catabolism.
• Our findings provide an explanation for RV vaccine failures in malnourished children.

## 1 Introduction

Rotavirus (RV) remains as a leading cause of childhood diarrhea worldwide. In 2000, RV caused an estimated 528,000 deaths in children under 5 years of age [1]. Due to global efforts since 2009, led by the World Health Organization (WHO) to introduce RV vaccines into routine childhood vaccination programs, estimated rotavirus deaths decreased to 146,000 in 2015 [2]. Vaccine efficacy against severe RV gastroenteritis was 85–98% in Latin American and European countries with mid to high socio-economic settings (SES). However, its efficacy was only 48–64% in African and Asian countries with low SES [3], [4]. Although lower RV vaccine efficacy in low SES countries is consistently reported, little is understood about the biologic mechanisms underlying the vaccine underperformance.

Because malnutrition is prevalent in African and south Asian countries with low RV vaccine efficacy, we hypothesized that malnutrition will affect the immune responses to oral attenuated human RV (AttHRV) vaccination and subsequent virulent HRV (VirHRV) challenge [5]. Indeed, some clinical studies reported low protection rates of RV vaccine against RV diarrhea in malnourished children [6], [7]. Malnutrition is a major contributor to the high mortality from viral gastroenteritis in low SES countries [4], [5], [8]. A number of field and animal studies have shown that malnutrition triggers immune dysfunction, including altered innate and adaptive immune responses, impairment of epithelial cell barriers and dysfunction of intestinal epithelial stem cells [9], [10], [11], [12], [13], [14]. However, studies to elucidate the effect of malnutrition on an oral AttHRV vaccine are lacking.

There is an increasing interest in understanding the complex interrelationships among nutrition, gut microbiota, host immunity and enteric pathogens. The gut microbiota, through their metabolites and components such as lipopolysaccharide (LPS), polysaccharide A and formyl peptides, contributes to the host physiological and immunological functions such as nutrient absorption, development and maturation of the gut immune system, and protection against exogenous pathogens [15], [16]. Nutrient availability impacts the composition and abundance of gut microbial taxa, and this in turn alters the nutritional metabolism of both the microbiota and the host, subsequently impacting the microbiota as well as host immunity and physiology [17], [18], [19]. Hence, physiologically relevant gnotobiotic (Gn) animal models that allow for nutritional and microbial manipulations are critical to assess and understand the interactions among nutrients, the microbiota and host immunity.

Tryptophan (TRP), an essential amino acid that cannot be synthesized de novo in humans, is reported to be a predictor of malnutrition [20], [21]. TRP also plays an important role in immune regulation. After its absorption via the neutral amino acid transport B^0^AT1 which is associated with angiotensin I converting enzyme 2 (ACE2), >90% of TRP is catabolized into kynurenine (KYN) via TRP 2,3-dioxygenase (TDO) in liver. However, upon infection and inflammation, extrahepatic TRP-KYN catabolism becomes dominant by inducing expression of indolamine 2,3-dioxygenase (IDO) on monocytes by stimulation with inflammatory cytokines such as IFN-α and IFN-γ [22], [23]. Local TRP depletion and production of KYN via IDO suppress proliferation of effector T cells and NK cells, but enhance regulatory T cell activities [24], [25], [26], [27]. IDO expression on dendritic cells (DCs) also induces further activation and differentiation of DCs [28]. Altered TRP homeostasis coinciding with decreased serum ACE2 levels has been observed previously in association with VirHRV challenge of Gn pigs fed a protein deficient diet [10]. However, the interrelationships between TRP-KYN metabolism and T cell and cytokine responses to RV vaccination and challenge are unclear.

We previously established a protein-deficient human infant fecal microbiota (HIFM)-transplanted neonatal Gn pig model that recapitulates major aspects of protein malnutrition in children [9], [10]. The aim of this study was to understand the immunologic and biologic mechanisms underlying the reduced vaccine efficacy in developing countries in the context of childhood protein deficiency. Here, using this HIFM-transplanted Gn pig model on protein sufficient or deficient bovine milk diet, we assessed the efficacy of the AttHRV oral vaccine against VirHRV challenge and compared innate, T cell and cytokine immune responses as well as TRP-KYN metabolism. We also included non-HIFM transplanted germ-free (GF) counterpart groups to elucidate the immunomodulating effects of the transplanted HIFM on protein deficiency and on the other study parameters.

## 2 Materials and Methods

### 2.1 Human infant fecal microbiota (HIFM)

The collection and use of HIFM were approved by The Ohio State University Institutional Review Board. With parental consent, sequential fecal samples were collected from a healthy, two-month-old, exclusively breastfed, vaginally delivered infant. Samples were pooled and diluted to 1:20 (wt/vol) in phosphate buffer solution containing 0.05% (vol/vol) cysteine and 30% glycerol and stored at −80 °C as described previously [9], [10].

### 2.2 Virus

The cell-culture adapted attenuated HRV (AttHRV) Wa G1P [8] strain passaged in African green monkey kidney cells (MA104) was used as a vaccine at a dose of 1 × 10^7^ fluorescent foci-forming units (FFU) [29]. The Gn pig passaged virulent HRV (VirHRV) Wa strain at pig passages 25–26 was used as challenge virus at a dose of 1 × 10^6^ FFU as described previously [9], [10].

### 2.3 Animal experiments

The animal experiments were approved by the Institutional Animal Care and Use Committee at The Ohio State University. Piglets were derived from near-term sows (purchased from OSU specific pathogen-free swine herd) by hysterectomy and maintained in sterile isolators [30]. Neonatal pigs obtained from five litters (5–15 pigs/litter) were randomly assigned to one of four groups: (1) protein deficient diet, GF (no HIFM) (Deficient group, n = 12); (2) protein sufficient diet, GF (no HIFM) (Sufficient group, n = 11); (3) protein deficient diet, HIFM transplanted (Deficient HIFM group, n = 12); and (4) protein sufficient diet, HIFM transplanted (Sufficient HIFM group, n = 11). Pigs in Sufficient GF and Sufficient HIFM groups were fed 100% ultra-high temperature pasteurized bovine milk (Parmalat) that met or exceeded the National Research Council Animal Care Committee’s guidelines for calories, fat, protein and carbohydrates in suckling pigs. Pigs in Deficient GF and Deficient HIFM groups were fed 50% Parmalat and 50% sterile water, which contained half of the recommended protein levels (7.5% vs 15% of diet). All pigs were confirmed as free from bacterial and fungal contamination prior to HIFM transplantation by aerobic and anaerobic cultures of rectal swabs. Pigs in Deficient HIFM and Sufficient HIFM groups were orally inoculated with 2 ml of diluted HIFM stock at 4 days of age (post-HIFM transplantation day, PTD 0). Rectal swabs were collected once or twice a week to analyze the microbial compositions by 16S metagenomic analysis as described previously [9], [31]. All pigs were orally vaccinated twice at a 10-days interval with AttHRV at PTD 7/post-1st vaccination day, PVD 0 and PTD 17/PVD 10 [post-2nd vaccination day 0, thereafter referred as PVD10 (0)]. At PTD 24/PVD 17 (7)/post-challenge day (PCD) 0, a subset of pigs from each of the four group was euthanized to assess vaccine responses pre-challenge. The remaining pigs were challenged with VirHRV and euthanized at PTD 31/PVD 24 (14) /PCD 7.

### 2.4 Assessment of clinical signs and detection of HRV shedding

Rectal swabs were collected daily post-challenge. Fecal consistency was scored as follows; 0, normal; 1, pasty/semi-liquid; and 2, liquid, and pigs with fecal score more than 1 were considered as diarrheic. Rectal swabs were suspended in 2 ml of minimum essential medium (MEM) (Life technologies, Waltham, MA, USA), clarified by centrifugation for 800*g* for 10 min at 4 °C, and stored at −20 °C until quantitation of infectious HRV by cell culture immunofluorescence assay as previously described [32].

### 2.5 Isolation of MNCs and flow cytometry

Blood, spleen, ileum and duodenum were collected to isolate mononuclear cells (MNCs) as described previously. Freshly isolated MNCs were stained for determining T cell subsets: T helper (CD3^+^CD4^+^) and cytotoxic T cells (CD3^+^CD8^+^), natural and inducible regulatory T cells (CD4^+^CD25^+^Foxp3^+^ and CD4^+^CD25^−^Foxp3^+^, respectively) and activated T cells (CD4^+^CD25^+^Foxp3^−^ and CD8^+^CD25^+^Foxp3^−^) [33]. To determine the frequencies of HRV-specific IFN-γ producing CD4^+^ and CD8^+^ cells, freshly isolated MNCs from spleen and ileum were re-stimulated *in vitro* with semi-purified AttHRV Wa strain (12 µg/ml) and porcine cross-reactive human CD49d mAb (0.5 µg/ml; clone 9F10, BD Pharmingen) for 18 h and stained as previously described [33], [34]. To assess frequencies of conventional DCs (cDCs, SWC3a^+^CD4^−^CD11R1^+^) and plasmacytoid DCs (pDCs, SWC3a^+^CD4^+^CD11R1^−^), MHC II and CD103 marker expression on DCs and TLR receptor expression on MNCs, cells were stained with monoclonal antibodies to porcine and human cell surface markers as previously reported [35], [36]. Data acquisition were done using MACSQuant Analyzer 10 (Miltenyi Biotech, San Diego, CA, USA) and analyses were conducted using FlowJo version 10 software (FLOWJO, LLC., Ashland, Oregon, USA).

### 2.6 NK cytotoxicity assay

Total spleen MNCs and K562 cells were used as effector and target cells, respectively. Effector: target cell ratios of 10:1, 5:1, 1:1 and 0.5:1 were used and the assay was done as described previously [37].

### 2.7 Detection of cytokines in serum and large intestinal contents (LIC) by ELISA

Blood samples were collected at multiple time points; PTD 0, PTD 7/PVD 0, PTD 9/PVD 2, PTD 17/PVD 10(0), PTD 19/PVD 9(2), PTD 24/PVD 17(7)/PCD 0, PTD 26/PVD 19 (9)/PCD 2 and PTD 31/PVD 24(14)/PCD 7. Serum and LIC samples were processed and analyzed for proinflammatory (TNF-α and IL-6), innate (IFN-α), Th1 (IL-12 and IFN-γ), Th2 (IL-4), and Treg (IL-10 and TGF-β) cytokines as described previously with some modifications [29], [33]. Briefly, Nunc Maxisorp 96-well plates were coated with anti-porcine IL-4 (2 µg/ml, clone A155B16F2), anti-porcine IL-10 (4 µg/ml, clone 945A4C437B1), anti-porcine IFN-γ (1.5 µg/ml, clone A151D5B8), anti-TGF-β (1.5 µg/ml, clone 55B16F2), (Thermo Fisher Scientific, Waltham, MA), anti-porcine IL-6 (0.75 µg/ml, goat polyclonal antibody), anti-porcine IL-12 (0.75 µg/ml, goat polyclonal antibody), anti-porcine IFN-α (2.5 µg/ml, clone K9) (R&D systems, Minneapolis, MN), or anti-porcine TNF-α (1.5 µg/ml, goat polyclonal antibody, Kingfisher biotech, Saint Paul, MN) overnight at 37 °C for IFN-α or 4 °C for the other cytokines. Biotinylated anti-porcine IL-4 (0.5 µg/ml, clone A155B15C6), anti-porcine IL-10 (1 µg/ml, clone 945A1A926C2), anti-porcine IFN-γ (0.5 µg/ml, clone A151D13C5), anti-TGF-β (0.4 µg/ml, TGF-F (0.4tispecies Antibody Pair, CHC1683) (Thermo Fisher Scientific, Waltham, MA), anti-porcine IL-6 (0.1 µg/ml, goat polyclonal IgG), anti-porcine IL-12 (0.2 µg/ml, goat polyclonal IgG), anti-porcine IFN-α (3.75 µg/ml, clone F17) (R&D systems, Minneapolis, MN), or anti-porcine TNF-α (0.4 µg/ml, goat polyclonal antibody, Kingfisher biotech, Saint Paul, MN) were used for detection. Porcine IFN-α detection antibody was biotinylated using a commercial kit as described previously [33]. Plates were developed and cytokine concentrations were calculated as described previously [29]. Sensitivities for these cytokines were 1 pg/ml for IL-4, IL-12 and IFN-α, 4 pg/ml for TNF-α, 8 pg/ml for TGF-β, and 16 pg/ml for IL-6, IL-10 and IFN-γ. Due to limited amounts, serum samples from PTD 0 to PTD 19/PVD 12(2) were only analyzed for IL-12, IFN-α, IFN-γ, TNF-α and TGF-β.

### 2.8 Quantification of serum tryptophan, kynurenine, angiotensin I converting enzyme 2 (ACE2) and endotoxin levels

Serum tryptophan, kynurenine, ACE2 and endotoxin concentrations were analyzed using commercially available ELISA kits according to manufacturer’s instructions (Tryptophan ELISA and Kynurenine ELISA, Rocky Mountain Diagnostics, Colorado Springs, CO; Porcine Angiotensin I Converting Enzyme 2 (ACE2) ELISA kit, AB clonal, Woburn, MA; and Pierce LAL Chromogenic Endotoxin Quantitation Kit, Pierce Biotechnology, Rockford, IL).

### 2.9 Statistical analysis

Differences in fecal scores and virus shedding titers amongst the groups were compared by one-way ANOVA followed by Dunn’s multiple comparison test. The absolute numbers of Th and Tc cells and frequencies of T cell subsets in flow cytometry, mean levels of serum cytokines, endotoxin, ACE2, tryptophan and kynurenine were compared between Deficient HIFM and Sufficient HIFM groups or between Deficient GF and Sufficient GF groups by Mann-Whitney U test. All statistical analyses were performed using GraphPad Prism version 7 (GraphPad software, Inc., La Jolla, CA). *P* values < 0.05 were considered statistically significant for all comparisons.

## 3 Results

### 3.1 Protein deficiency decreased protection rates of oral AttHRV vaccine against diarrhea and resulted in prolonged and high titer of virus shedding post-challenge in HIFM transplanted pigs

The oral AttHRV vaccine protected all pigs in the Sufficient HIFM group from diarrhea after VirHRV challenge, whereas 33.3% of pigs in Deficient HIFM group had mild diarrhea with a mean duration of 1.2 days (Table 1). The Deficient HIFM group had higher mean cumulative fecal scores from PCD 1 to PCD 6 (5.3 versus 3.8, Table 1) and a significantly higher mean fecal score at PCD 4 (*p* < 0.01) compared with the Sufficient HIFM group (data not shown). Higher fecal scores in the Deficient HIFM group compared with the Sufficient HIFM group coincided with a higher rate of virus shedding (100% versus 33.3%, Table 1), significantly higher geometric mean of peak HRV titers shed (797.4 versus 35.4 FFU/ml, Table 1, *p* < 0.001) and longer mean duration of virus shedding (2.5 versus 1.5 days, Table 1) compared with the Sufficient HIFM group.

**Table 1:** Summary of diarrhea and fecal VirHRV shedding after VirHRV challenge (PCD 1 to PCD 6).

Group^a^	*N*	Diarrhea^b^			Virus shedding^c^		
		% of pigs	Mean cumulative fecal score^d^	Mean duration (days)^e^	% of pigs	Geometric mean of peak titer (FFU/ml)^f^	Mean Duration (days)
Deficient HIFM	6	33.3	5.3	1.2	100	797.4^**^	2.5
Sufficient HIFM	6	0.0	3.8	NA	33.3	35.4^*^	1.5

a Deficient HIFM; gnotobiotic (Gn) pigs transplanted with human infant fecal microbiota (HIFM) at 4 days of age (PTD 0) and fed protein deficient diet, Sufficient HIFM; Gn pigs transplanted with HIFM at 4 days of age and fed protein sufficient diet, All pigs were orally vaccinated twice with attenuated human rotavirus Wa strain (AttHRV) on post-transplantation day (PTD) 7 and 17, and challenged with virulent human rotavirus (VirHRV) on PTD 24.

b Pigs with fecal score > 1 were considered as diarrheic. Fecal consistency was scored as follows: 0, normal; 1, pasty; 2, semi-liquid; and 3, liquid.

c Determined by cell culture immunofluorescence assay and expressed as FFU/ml.

d Mean of total of fecal scores from PCD 1 to PCD 6.

e Mean of the total days with fecal score > 1.

f Samples negative for HRV detection (<25) were assigned a titer of 12.5 for statistical analysis. Means in the same column with different asterisks differ significantly

In the GF counterpart groups, the AttHRV vaccine also gave better protection against diarrhea post-challenge in Sufficient GF pigs compared with Deficient GF pigs (50% vs 33.3%, data not shown). However, the prevalence of virus shedding, peak titer and the mean duration of virus shedding post-challenge in Deficient GF pigs (33.3%, 53.7 FFU/ml and 1.0 day, data not shown) were almost equivalent to those from Sufficient pigs (33.3%, 46.5 FFU/ml and 1.0 day, data not shown) and the Sufficient HIFM pigs (33.3%, 35.4 FFU/ml and 1.5 days, Table 1), but significantly lower than in Deficient HIFM pigs, suggesting that HIFM transplantation exacerbated the negative effects of protein deficiency on the HRV vaccine efficacy.

### 3.2 Protein deficiency resulted in suppression of multiple innate immune parameters

**Fig. 1:**
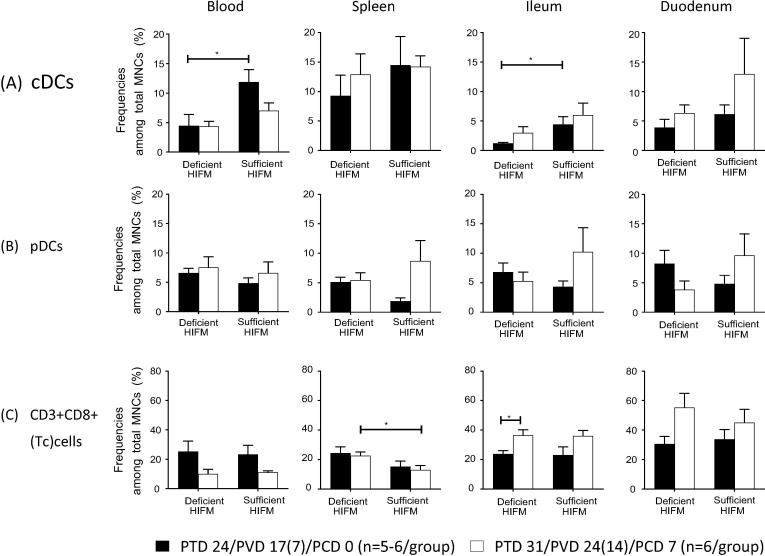
Mean frequencies (±SEM) of (A) conventional dendritic cells (cDCs), (B) plasmacytoid dendritic cells (pDCs), and (C) cytotoxic T cells. Significant difference (*p < 0.05) between HIFM transplanted gnotobiotic pigs fed protein deficient diet (Deficient HIFM) and protein sufficient diet (Sufficient HIFM) groups or between pre- and post-virulent HRV challenge in each group were determined by Mann-Whitney U test. HRV; human rotavirus, Deficient; protein deficient diet, Sufficient; protein sufficient diet, HIFM; human infant fecal microbiota, PTD; post transplantation day, PVD; post 1st (2nd) vaccination day and PCD; post challenge day.

Deficient HIFM pigs had significantly lower frequencies of conventional dendritic cells (cDCs) in blood and ileum pre-challenge compared with Sufficient HIFM pigs (*p* < 0.05) (Fig. 1A). Although increased cDC frequencies were observed post-challenge in ileum and duodenum of both Deficient HIFM and Sufficient HIFM pigs, decreased frequencies in blood were apparent only in Sufficient HIFM pigs (Fig. 1A). Interestingly, Deficient HIFM pigs showed sustained or decreased frequencies of plasmacytoid dendritic cells (pDCs) post-challenge in spleen, ileum and duodenum (0.5- to 1.1-fold change) in contrast to Sufficient HIFM pigs in which those frequencies increased by 2.0- to 4.6-fold in the respective tissues (Fig. 1B). Similar trends, but without statistically significant differences, were observed in the above immune parameters of the GF counterpart groups (Supplemental Fig. 1). No effects of protein deficiency were observed on frequencies of MHC class II and CD103 expressing pDCs and cDCs in blood and each tissue between Deficient HIFM and Sufficient HIFM groups as well as Deficient GF and Sufficient GF groups (data not shown).

**Fig. 2:**
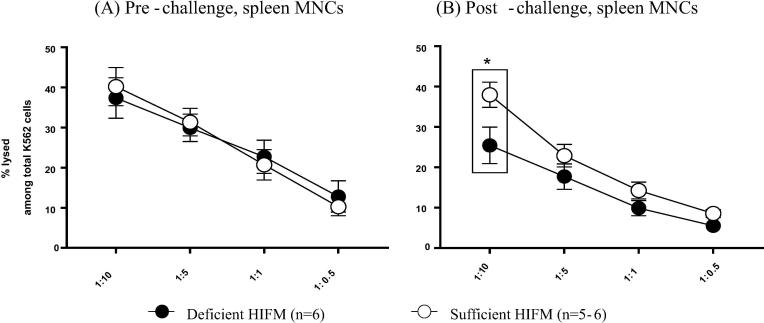
NK cell function of spleen MNCs in HIFM transplanted gnotobiotic pigs fed protein deficient diet (Deficient HIFM) and protein sufficient diet (Sufficient HIFM) groups (A) pre- and (B) post-challenge with virulent HRV. Spleen MNCs were co-cultured with carboxyfluorescein succinimidyl ester (CFSE)-stained K562 cells at indicated ratios overnight. The dead cells were stained by incubating with 7-aminoactinomycin D (7-AAD). The cells were observed by flowcytometry to obtain % lysed K562 cells among total K562 cells. Significant differences (*p < 0.05) between Deficient HIFM and Sufficient HIFM groups were determined by Mann-Whitney U test. HRV; human rotavirus, Deficient; protein deficient diet, Sufficient; protein sufficient diet, HIFM; human infant fecal microbiota.

The frequencies of natural killer (NK) cells in blood and the tissues were not significantly different between Deficient HIFM and Sufficient HIFM pigs both pre- and post-challenge (data not shown). However, the cytotoxic function of NK cells was consistently lower and significantly reduced in MNCs from spleen of Deficient HIFM pigs post-challenge (but not pre-challenge) compared with Sufficient HIFM pigs (Fig. 2). Similar suppression in the cytotoxic function of NK cells post-challenge was also observed in the Deficient GF pigs (Supplemental Fig. 2).

### 3.3 Frequencies of TLR2 and TLR4 expressing MNCs were differentially affected by protein deficiency in HIFM transplanted pigs

**Fig. 3:**
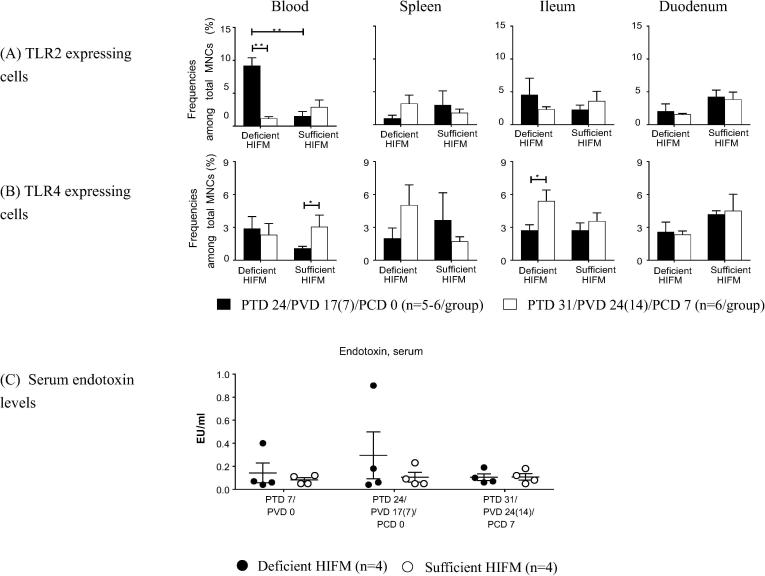
Mean frequencies (±SEM) of MNCs expressing (A) TLR2 and (B) TLR4, and (C) serum endotoxin levels in HIFM transplanted gnotobiotic pigs fed protein deficient diet (Deficient HIFM) and protein sufficient diet (Sufficient HIFM) groups. Mononuclear cells (MNCs) were isolated from blood, spleen, ileum, and duodenum of the piglets from Deficient HIFM and Sufficient HIFM groups pre- and post-virulent HRV challenge (PTD 24/PCD 0 and PTD 31/PCD 7, respectively). The mean serum endotoxin levels are shown with SEM. Significant difference (*p < 0.05) between Deficient HIFM and Sufficient HIFM groups or between pre- and post-virulent HRV challenge in each group were determined by Mann-Whitney U test. HRV; human rotavirus, Deficient; protein deficient diet, Sufficient; protein sufficient diet, HIFM; human infant fecal microbiota, PTD; post transplantation day, PVD; post 1st (2nd) vaccination day and PCD; post challenge day.

The effect of protein deficiency on expression of TLR2 (ligands-bacterial peptidoglycan, lipoteichoic acids and lipoproteins), TLR4 (ligand-bacterial lipopolysaccharide, LPS), TLR9 (ligand-bacterial CpGs), and TLR3 (ligand-double stranded RNAs) were analyzed for MNCs from blood and the tissues (Fig. 3).

Frequencies of TLR2 expressing MNCs were significantly higher in blood of Deficient HIFM pigs pre-challenge as compared with Sufficient HIFM pigs, and significantly decreased post-challenge (Fig. 3A). Similar trends were also observed in the blood of GF counterpart groups, except that Sufficient pigs had significantly increased frequencies of TLR2 expressing MNCs in duodenum post-challenge as compared with pre-challenge as well did Deficient pigs post-challenge (Supplemental Fig. 3).

Frequencies of TLR4 expressing MNCs did not differ significantly between Deficient HIFM and Sufficient HIFM pigs (Fig. 3B). However, significantly increased frequencies of TLR4 expressing MNCs were observed in blood of Sufficient HIFM pigs and ileum of Deficient HIFM pigs post-challenge (Fig. 3B). These trends and fluctuations observed in the HIFM transplanted groups were not observed in the GF counterpart groups, and Deficient GF pigs had significantly lower frequencies of TLR4 expressing MNCs in spleen pre-challenge as compared with Sufficient GF pigs (Supplemental Fig. 3). Serum endotoxin (LPS) levels were low in Sufficient HIFM pigs at PTD 7/PVD 0, PTD24/PVD17(7)/PCD 0 and PTD 31/PVD24 (14)/PCD 7(0.05–0.23 EU/ml, Fig. 3C). A similar trend was observed in the Deficient HIFM pigs. However, two different pigs had increased serum LPS levels either at PTD 7/PVD 0 or PTD24/PVD17(7)/PCD 0 (Fig. 3C). There were no consistent effects of protein deficiency or HIFM transplantation on the expression of TLR3 and TLR9 both pre- and post-challenge (data not shown).

### 3.4 Protein deficiency significantly increased frequencies of CD3^+^CD8^+^ (cytotoxic T, Tc) cells, but significantly decreased frequencies of HRV-specific CD3^+^CD4^+^ IFN-γ producing T cells post-challenge in the HIFM transplanted pigs

Deficient HIFM pigs had significantly higher frequencies of Tc cells in spleen post-challenge compared with that in Sufficient HIFM pigs (*p* < 0.05), and significantly increased frequencies of Tc cells in the ileum post-challenge compared with pre-challenge (*p* < 0.05) (Fig. 1C). Increased Tc cell frequencies were also observed in the ileum of Sufficient HIFM pigs and duodenum of both Deficient HIFM and Sufficient HIFM pigs (Fig. 1C) as well as that of the GF counterpart groups (Supplemental Fig. 1), although not significant. No effect of protein deficiency was evident on the frequencies of CD3^+^CD4^+^ (T helper, Th) cells pre- and post-challenge between Deficient HIFM and Sufficient HIFM groups as well as Deficient GF and Sufficient GF groups (data not shown).

**Fig. 4:**
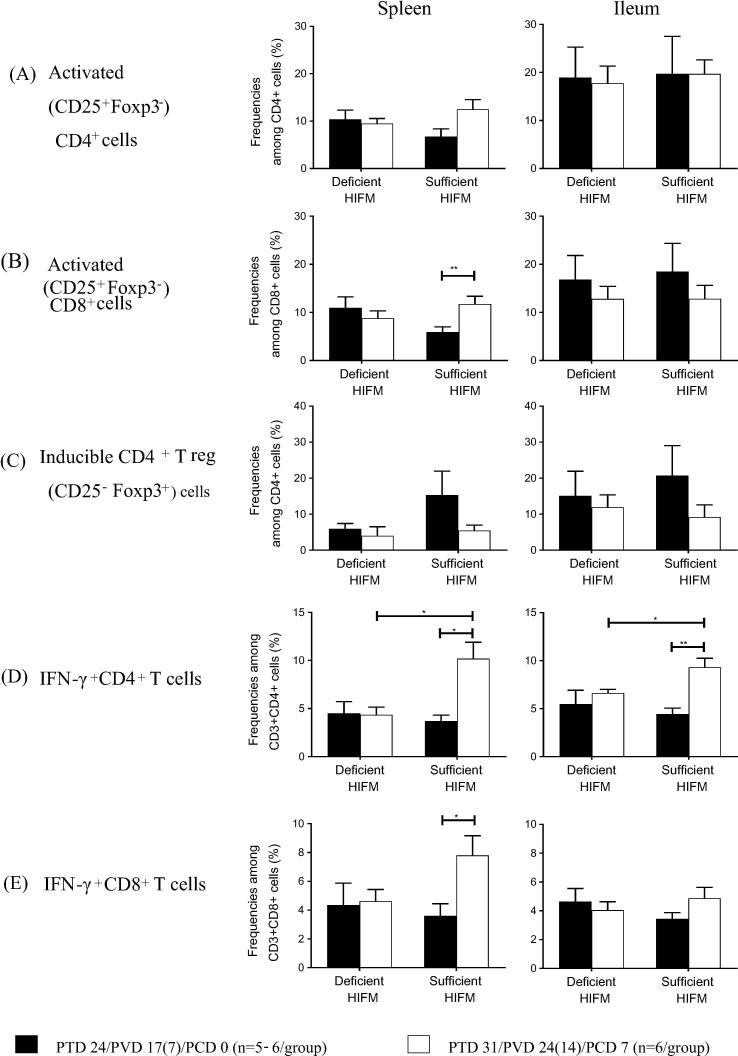
Mean frequencies (±SEM) of activated (CD25^+^Foxp3^−^) cells among CD4^+^ cell (A) and CD8^+^ cell (B) subsets, and inducible T regulatory (CD25^−^Foxp3^+^) cells among CD4^+^ cell subsets (C) and HRV specific IFN-γ producing cells among CD3^+^CD4^+^ (D) and CD3^+^CD8^+^ (E) T cell subsets. Significant difference (*p < 0.05, **p < 0.01) between HIFM transplanted gnotobiotic pigs fed protein deficient diet (Deficient HIFM) and protein sufficient diet (Sufficient HIFM) groups or between pre- and post-virulent HRV challenge in each group were determined by Mann-Whitney U test. HRV; human rotavirus, Deficient; protein deficient diet, Sufficient; protein sufficient diet, HIFM; human infant fecal microbiota, HIFM; human infant fecal microbiota, PTD; post transplantation day, PVD; post 1st (2nd) vaccination day and PCD; post challenge day.

Sufficient HIFM pigs had increased frequencies of activated (CD25^+^Foxp3^−^) CD4^+^ (1.8-fold, numerically) and CD8^+^ cells (2.0-fold, significant, *p* < 0.05) in spleen post-challenge as compared with pre-challenge (Fig. 4A and B). In contrast, those trends were not observed in spleen of Deficient HIFM pigs post challenge (Fig. 4A and B). The trends in the fluctuation of frequencies of activated T cells in ileum post-challenge were generally similar between Deficient HIFM and Sufficient HIFM groups. Frequencies of inducible (CD25^−^Foxp3^+^) CD4^+^ regulatory T (Treg) cells decreased post-challenge in spleen and ileum of Sufficient HIFM pigs (0.4-fold), but they remained stable in Deficient HIFM pigs post-challenge (Fig. 4C). Similar trends were also noted for the GF counterpart groups (Supplemental Fig. 4).

Coinciding with the higher protection rate against virus shedding and shorter duration, Sufficient HIFM pigs had significantly increased frequencies of HRV-specific IFN-γ^+^ CD4^+^ T cells in spleen and ileum and HRV-specific IFN-γ^+^ CD8^+^ T cells in spleen after VirHRV challenge compared with pre-challenge (Fig. 4D and E). In contrast, the frequencies of HRV specific IFN-γ^+^ T cells remained unchanged post-challenge in both the spleen and ileum of Deficient HIFM pigs and were significantly lower post-challenge than in the Sufficient HIFM pigs (Fig. 4D and E). In contrast, in the GF counterparts, pigs in both Deficient GF and Sufficient GF groups had increased frequencies of HRV specific IFN-γ^+^ CD4^+^ and CD8^+^ T cells in the spleen and in the ileum, except for those in the ileum of Sufficient GF pigs (Supplemental Fig. 4).

### 3.5 Protein deficiency compromised innate, proinflammatory and Th 1 cytokine responses after oral AttHRV vaccination as well as after VirHRV challenge in HIFM transplanted pigs

**Fig. 5:**
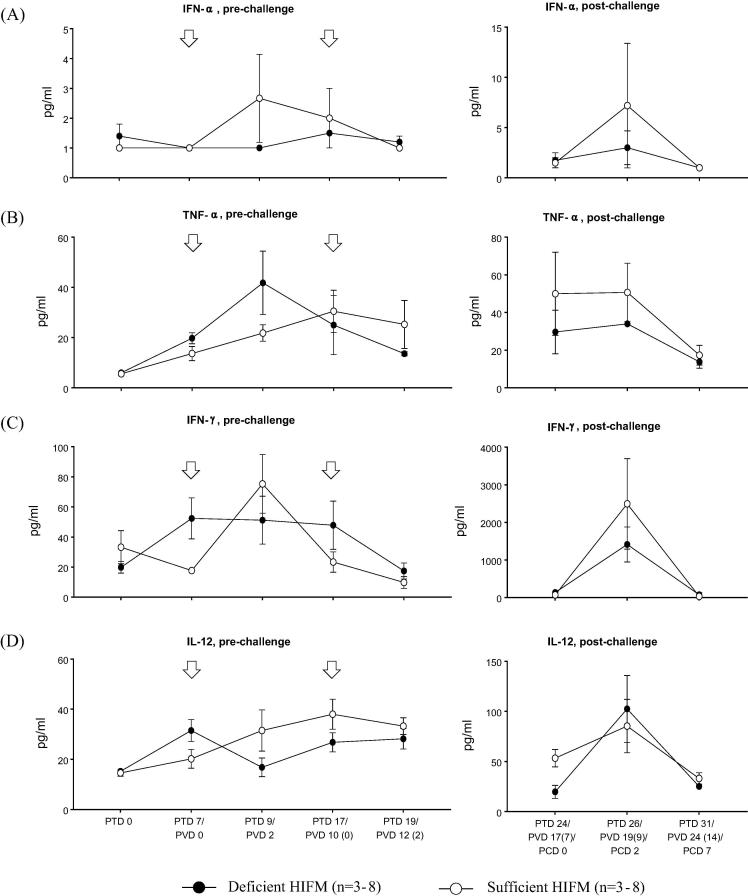
Mean concentrations (±SEM) of innate (IFN-α), proinflammatory (TNF-α), Th1 (IFN-γ and IL-12) and T regulatory (TGF-β) cytokines in serum of HIFM transplanted gnotobiotic pigs fed protein deficient diet (Deficient HIFM) and protein sufficient diet (Sufficient HIFM) groups before and after virulent human rotavirus challenge. White arrows indicate time points for oral inoculation of attenuated HRV. Significant difference (*p < 0.05 and **p < 0.01) between Deficient HIFM and Sufficient HIFM groups at each time point were determined by Mann-Whitney U test. HRV; human rotavirus, Deficient; protein deficient diet, Sufficient; protein sufficient diet, HIFM; human infant fecal microbiota, PTD; post transplantation day, PVD; post 1st (2nd) vaccination day and PCD; post challenge day.

We next compared the serum levels of innate (IFN-α), proinflammatory (TNF-α), Th1 (IFN-γ and IL-12), and Treg (TGF-β) cytokines in serum of Deficient HIFM and Sufficient HIFM groups (Fig. 5). In Sufficient HIFM pigs, serum IFN-α and IFN-γ concentrations peaked two days after the first vaccination (PTD 9/PVD 2) and decreased to the baseline levels by two days after the second vaccination [PTD 19/PVD 12 (2)]. Similarly, TNF-α and IL-12 increased gradually after the first vaccination and peaked on PTD 17/PVD 10 (0) with no increases after the second vaccination. In contrast in the Deficient HIFM pigs, the AttHRV vaccine failed to induce increases in the serum levels of IFN-α, IFN-γ and IL-12 and only enhanced serum pro-inflammatory TNF-α responses. However, the Deficient HIFM pigs already had numerically higher serum levels of IFN-γ and IL-12 on PTD 7/PVD 0 (on the day first vaccine dose was administered) as compared with the Sufficient HIFM pigs. Serum IFN-γ levels were maintained until PTD 17/PVD 10(0) without any further augmentation by the second vaccine dose in Deficient HIFM group. Serum IL-12 levels dropped after the first vaccination (PTD 9/PVD 2) in the Deficient HIFM group but then gradually increased at PTD 19/PVD 12 (2). However, serum IL-12 concentrations in the Deficient HIFM group were lower than those in the Sufficient HIFM pigs from PTD 9/PVD 2 to PTD 19/PVD 12 (2). These altered IFN-α, IFN-γ and IL-12 responses were observed only in Deficient HIFM pigs, but not in the Deficient GF pigs, suggesting the combined effects of HIFM transplantation and protein deficiency (Supplemental Fig. 5). Serum TGF-β responses pre-challenge were similar between Deficient HIFM and Sufficient HIFM groups (data not shown).

The overall trends of serum cytokine responses post-challenge were similar between Deficient HIFM and Sufficient HIFM groups: IFN-α, TNF-α, IFN-γ and IL-12 increased on PCD2 and decreased to baseline levels or lower (TNF-α and IL-12) by PCD 7 (Fig. 5). However, the Deficient HIFM group had numerically lower levels of serum TNF-α and IL-12 on PCD 0, and TNF-α, IFN-α and IFN-γ on PCD 2. These low responses of proinflammatory and Th1 cytokines post-challenge in the Deficient HIFM group were also observed in the Deficient GF group that shed less virus post-challenge (Supplemental Fig. 5). No significant differences were observed post-challenge for other proinflammatory (IL-6), Th 2 (IL-4) and Treg (IL-10 and TGF-β) cytokines both in serum and large intestinal contents (LIC) between Deficient HIFM and Sufficient HIFM groups as well as the Deficient GF and Sufficient GF groups (data not shown).

### 3.6 Deficient HIFM pigs maintained lower serum KYN levels throughout the study as compared with Sufficient HIFM pigs

**Fig. 6:**
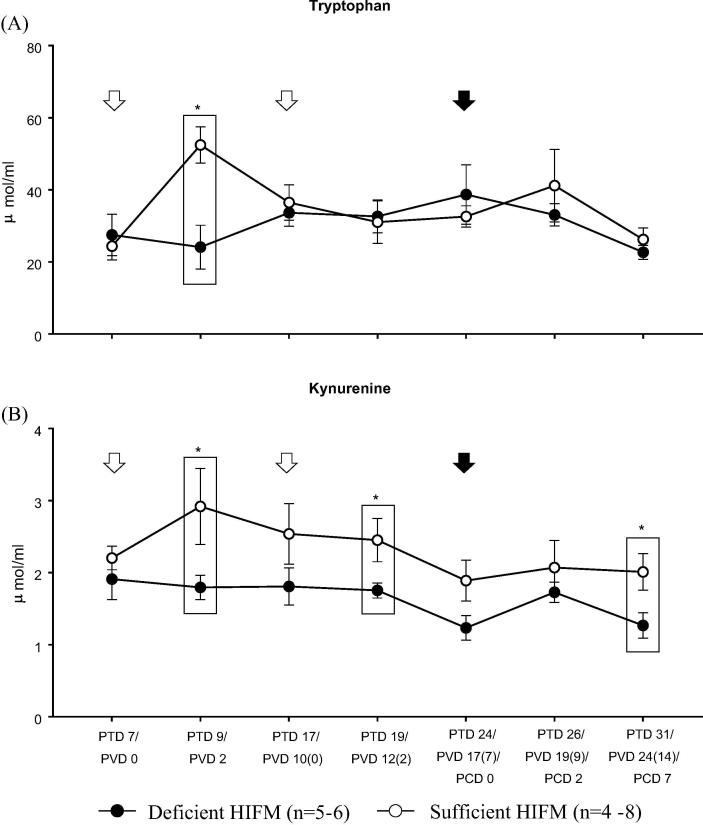
Mean concentrations (±SEM) of tryptophan and kynurenine in serum of HIFM transplanted gnotobiotic pigs fed protein deficient diet (Deficient HIFM) and protein sufficient diet (Sufficient HIFM) groups. White arrows and a black arrow indicate time points for vaccination by oral attenuated HRV inoculation and virulent HRV challenge, respectively. Significant difference (*p < 0.05) between Deficient HIFM and Sufficient HIFM groups at each time point were determined by Mann-Whitney U test. HRV; human rotavirus, Deficient; protein deficient diet, Sufficient; protein sufficient diet, HIFM; human infant fecal microbiota, PTD; post transplantation day, PVD; post 1st (2nd) vaccination day and PCD; post challenge day.

No significant differences were observed in serum ACE2 concentrations between Deficient HIFM and Sufficient HIFM groups throughout the study, suggesting pigs in both groups had the capability to absorb/transport TRP via the ACE2/B^0^AT1 transport pathway at a similar level (data not shown). However, as compared with the Sufficient HIFM groups, the Deficient HIFM group had significantly lower levels of serum TRP and KYN two days after the first vaccination (PTD 9/PVD 2) (Fig. 6). No significant differences were observed in serum TRP levels thereafter between the two groups. However, the Deficient HIFM group maintained lower serum KYN levels as compared with the Sufficient HIFM group until the end of the study with significant differences at PTD 19/PVD12 (2) and PTD 31/PVD 24 (14)/PCD 7. In contrast, those differences in serum TRP and KYN levels were not evident between the GF counterpart groups (Supplemental Fig. 6), once again suggesting the combined impact of HIFM transplantation with protein deficiency.

## 4 Discussion

Here, using a HIFM transplanted Gn pig model, we showed that protein deficiency impairs multiple aspects of innate, T cell and cytokine immune responses and reduces the efficacy of the oral AttHRV vaccine. Interestingly, in protein deficient pigs, more prolonged and higher titers of virus shedding after VirHRV challenge was evident only in HIFM transplanted pigs, but not in GF pigs, suggesting that transplanted HIFM exacerbated the negative impact of protein deficiency on HRV immunity and virus shedding. Our findings suggest that prolonged virus shedding from vaccinated but unprotected malnourished children could result in continuous circulation of RV in the community and co-circulation of genotypically different strains, which increase the risk of RV infections and increased RV diarrhea burden among infants and children in these communities.

Innate immune responses are critical as a first line of defense, limiting rotavirus replication and disease in the host [38], [39]. Type I interferons (IFN-α/β) produced by HRV-infected intestinal epithelial cells induce an anti-virus status in uninfected surrounding cells [39]. The importance of this DC-IL-12-NK cell innate immune axis is well documented in RV infections [9], [38], [40]. In the present study, protein deficiency resulted in suppression of multiple innate immune parameters involved in the DC-IL-12-NK immune axis, that was most pronounced in pigs transplanted with HIFM. First, we observed suppressed IFN-α and IL-12 responses after oral AttHRV vaccination in Deficient HIFM pigs, which could result in failure to promote subsequent innate and adaptive immunity. Indeed, the Deficient HIFM pigs had significantly lower frequencies of cDCs in blood and ileum as well as lower serum IL-12 and TNF-α levels (pre-challenge) and lower serum IFN-α and TNF-α levels (post-challenge) as compared with Sufficient HIFM pigs. These coincided with failure to increase the frequencies of pDCs (potent IFN-α producers) and decreased NK cell function post-challenge. Suppressed innate immune parameters in the Deficient HIFM pigs likely resulted in the failure to increase frequencies of IFN-γ producing T cells and to decrease the frequencies of inducible Treg cells post-challenge. Previous studies have shown that the protection rates against RV challenge correlate positively with HRV-specific IFN-γ producing T cell responses, but negatively with those of inducible Treg (CD4^+^CD25^−^Foxp3^+^) cells which are derived from naïve CD4^+^CD25^−^ T cells in response to rotavirus infection and vaccination [41], [42], [43], [44]. Collectively, the decreased vaccine efficacy observed in the Deficient HIFM pigs could be attributed to a cascade wherein the initial impairment of the cytokine responses pre-AttHRV vaccination contributed to the failure to induce balanced responses of effector and regulatory T cell responses after AttHRV vaccination and VirHRV challenge.

Protein deficiency also altered TRP-KYN metabolism in pigs transplanted with HIFM (Fig. 6). The half-life of an apoenzyme of TDO is extremely short and only TRP can stabilize TDO activity [23]. Thus, we conclude that the increase of serum KYN levels after the first AttHRV dose in Sufficient HIFM pigs (PTD 9/PVD 2, Fig. 6) could be due to AttHRV stimulation of IFN-α and IFN-γ production leading to enhanced IDO expression, which mediates TRP catabolism and increased serum KYN. The increased TRP catabolism might then down-regulate TDO activity in liver which in turn may result in increased serum TRP levels. In contrast, Deficient HIFM pigs had sustained levels of serum KYN and TRP which coincided with the reduced serum IFN-α and IFN-γ responses after AttHRV vaccination and VirHRV challenge. Serum levels of KYN in Deficient pigs were significantly lower than those of Sufficient HIFM pigs, suggesting that the altered TRP-KYN metabolism in the Deficient HIFM pigs could be a result of, but also contribute to the suppressed IFN responses. TRP-KYN metabolism is known to affect T cell immunity; specifically, it converts local T cell function from an immunogenic one to a tolerogenic one [23], [45]. Indeed, increased frequencies of HRV specific IFN-γ producing T cells and decreased frequencies of inducible CD4^+^ Treg cells were observed post-challenge in the Sufficient HIFM pigs, but not in the Deficient HIFM pigs (Fig. 5). Collectively, the altered TRP-KYN metabolism could also directly or indirectly contribute to the altered T cell responses and accordingly the prolonged and high titer of virus shedding which were observed in the Deficient HIFM pigs.

Proliferation and function of DCs, T and NK cells depend on nutrient availability (such as amino acids and glucose) which modulates growth signals and protein synthesis rates of these cells via the glycolytic pathway [26], [46], [47]. Our previous study demonstrated hypoglycemia and hypoproteinemia in HIFM transplanted Gn pigs fed the same protein deficient diet in association with altered (generally down-regulation) immune responses [9]. These factors may also contribute to the defects in innate, T cell and cytokine immune responses as well as the differential TLR2 and TLR4 expression observed in MNCs in Deficient HIFM pigs in our present study. Although most of the changes in the immune parameters in the Deficient HIFM pigs were also observed in the GF counterparts, the duration and the peak titer of virus shedding post-VirHRV challenge did not differ between the Deficient GF pigs and the Sufficient HIFM or GF pigs, suggesting that the defects in immune parameters were aggravated by HIFM transplantation. In addition, our observations of the increased serum levels of IFN-γ and TNF-α (that affect epithelial integrity [48], [49]) in Deficient HIFM pigs suggest that, similar to the findings in our previous study [9], the Deficient HIFM pigs likely had intestinal epithelial barrier dysfunction starting before the AttHRV vaccination. Intestinal epithelial damage would further exacerbate the malabsorption and trigger the translocation of gut microbiota as suggested by the significantly higher frequency of TLR2 (which recognizes bacterial lipoprotein and peptide glycan of Gram positive bacteria) expressing cells in blood of the Deficient HIFM pigs as compared with Sufficient HIFM pigs pre-challenge (Fig. 3A). In addition, similar to our previous observations on the increased serum LPS levels that coincided with impaired intestinal epithelial barrier in protein-deficient pigs [9], [31], in the present study we observed elevated serum endotoxin levels before and after AttHRV vaccination in some Deficient HIFM pigs (Fig. 3C). Hughes et al. reported defective functions of DCs from severely malnourished children with endotoxemia, including absent or low IL-12 production, down-regulation of HLA-DR and inability to support T cell proliferation [12], which were similar to our findings of the decreased IL-12 production and lack of activated Th and Tc cell responses in Deficient HIFM pigs. Collectively, these findings suggest that the defects in innate immune responses in the Deficient HIFM pigs might be triggered by protein deficient diet and aggravated by direct competition for nutrients by the HIFM and translocation of gut microbiota.

Analysis of the detailed microbiota data from this study will be addressed in a subsequent paper (Srivastava et al., unpublished). In brief, protein deficiency decreased the *Firmicutes*- to -*Bacteroides* ratios post-challenge in the HIFM transplanted pigs, which coincided with increased *Proteobacteria* levels (mainly *Proteus* genus) while decreased *Firmicutes* levels (mainly *Turicibacter* genus) occurred in spleen and ileum. Relative abundance of *Turicibacter* is a good indicator of mice with a functioning immune system, and decreased abundance of *Turicibacter* is associated with reduction in multiple immune cells [50], [51]. While these alterations in the intestinal microbial profiles coincided with more pronounced effects of protein deficiency (enhanced VirHRV shedding and diarrhea) in protein deficient HIFM transplanted as compared with GF pigs, these findings are not sufficient for establishing a causal relationship. Moreover, although more pronounced in the HIFM transplanted protein deficient pigs, the changes in the immune responses were similar to those in GF protein deficient pigs. It may be challenging to reliably link most of the observed changes in immune responses to the altered abundance of certain commensal species because we previously demonstrated that the protein deficiency induced numerous changes in the host microbiome composition without evidence that these changes alone could induce suppression of different immune responses [9], [31]. There is limited data on mechanisms by which the gut microbiota can influence immunity. Bacterial components including LPS, polysaccharide A and formyl peptides have been shown to impact innate immunity [15], [17], [52] and could also impact the host cell barrier functions by modifying surface glycan and/or galactose, as well as by interfering with virus infection by direct binding [53], [54]. In our future studies, additional analyses such as metabolomics and transcriptome analysis will help to elucidate the underlying mechanisms of the immune impairment of RV vaccine responses triggered by protein deficiency and the altered microbiota.

In conclusion, using HIFM transplanted and GF neonatal pig models, we demonstrated that protein deficiency impairs the efficacy of an oral AttHRV vaccine by altering innate, T cell and cytokine immune responses. There is a growing interest in delineating the interactions among nutrition, metabolites, microbiota and host immunity and the impacts of those interactions on infection and vaccine immunity.

Our findings on the associations between the impaired immunity and altered TRP-KYN catabolic pathway provide novel insights and identify possible predictors of AttHRV efficacy that could be applicable to children. The HIFM transplanted neonatal pig model can be further used for mechanistic evaluation of the effects of physiologically relevant interventions.

## Conflict of interest

The authors declare no conflict of interest.

## Author contribution

Conceived and designed the experiments: GR, LJS and AV. Data collection: AM, SK, HM, SNL, FCP, JC, MAA, DDF, HCH, VS, DK and LC. Analyzed the data: AM, SK, HM and LD. Wrote the paper: AM.
