# Supplementary material for: Protein deficiency reduces efficacy of oral attenuated human rotavirus vaccine in a human infant fecal microbiota transplanted gnotobiotic pig model
Source: Vaccine. 2018 Oct 8;36(42):6270–81. doi: 10.1016/j.vaccine.2018.09.008 (PMC6180620; doi:10.1016/j.vaccine.2018.09.008)
Supplement: Supplementary data 1 [file mmc1.pptx]

## Slide 1
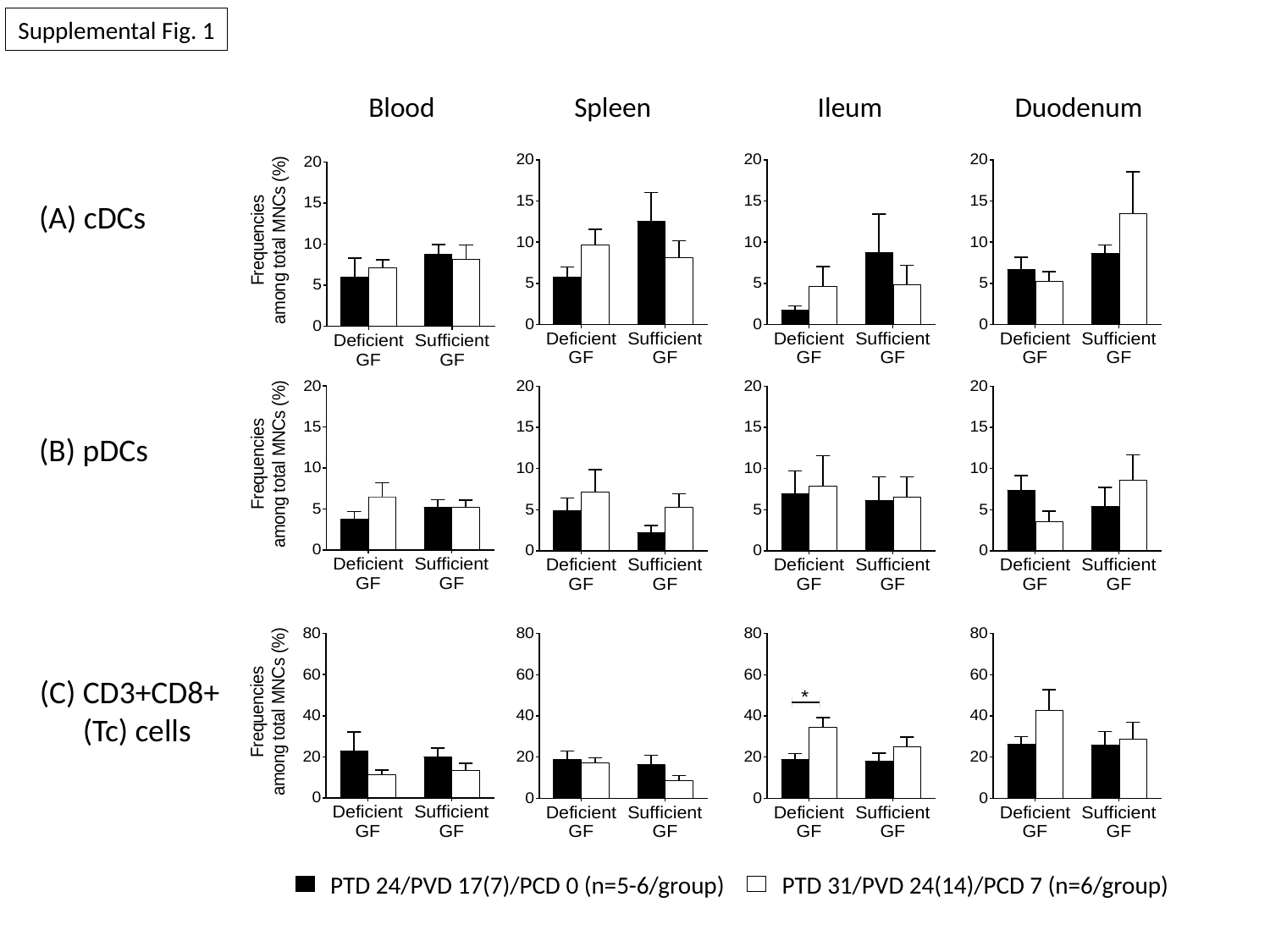

Supplemental Fig. 1
Blood
Spleen
Ileum
Duodenum
(A) cDCs
(B) pDCs
(C) CD3+CD8+
 (Tc) cells
PTD 24/PVD 17(7)/PCD 0 (n=5-6/group)
PTD 31/PVD 24(14)/PCD 7 (n=6/group)

## Slide 2
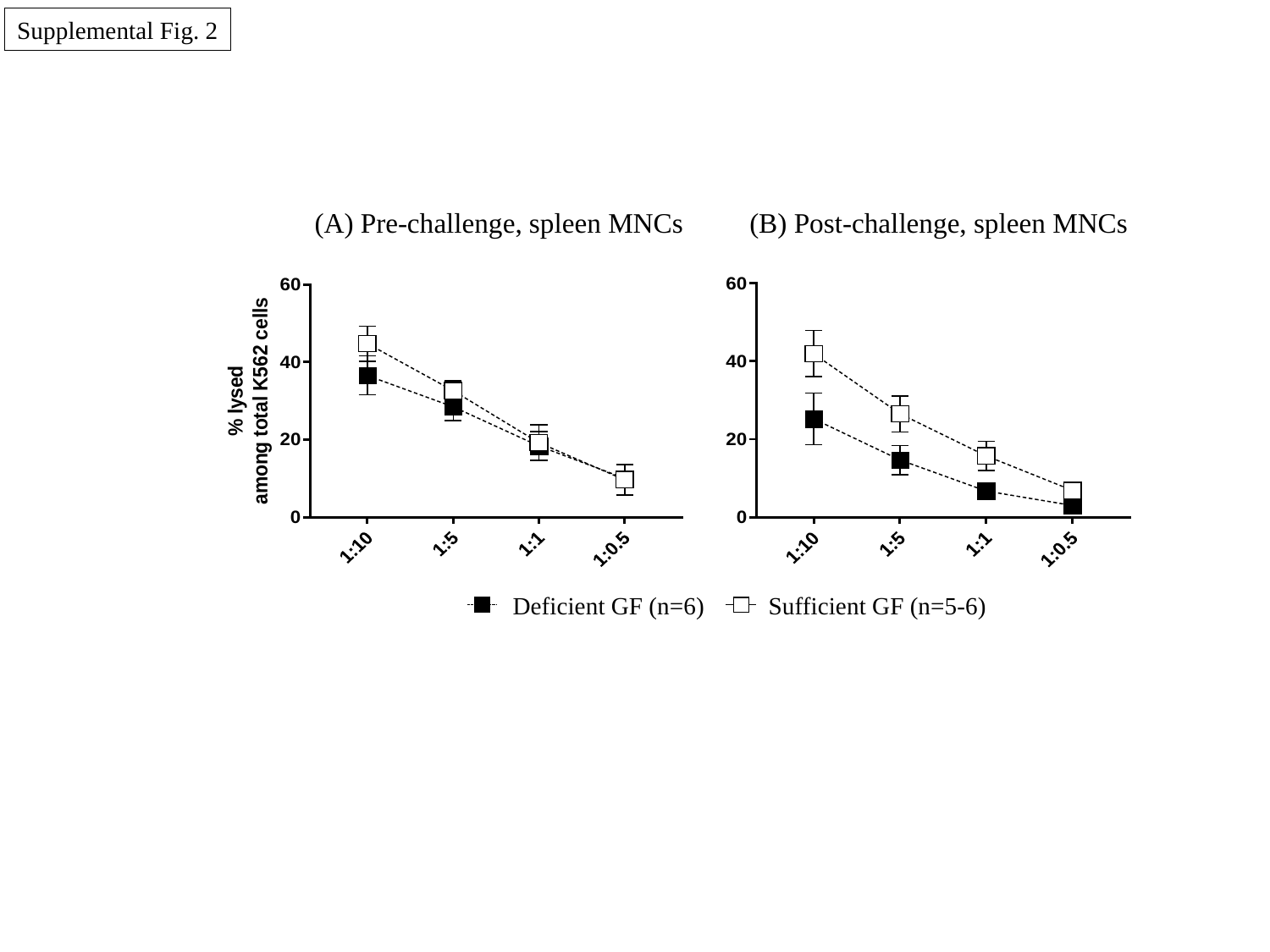

Supplemental Fig. 2
(A) Pre-challenge, spleen MNCs
(B) Post-challenge, spleen MNCs
Deficient GF (n=6)
Sufficient GF (n=5-6)

## Slide 3
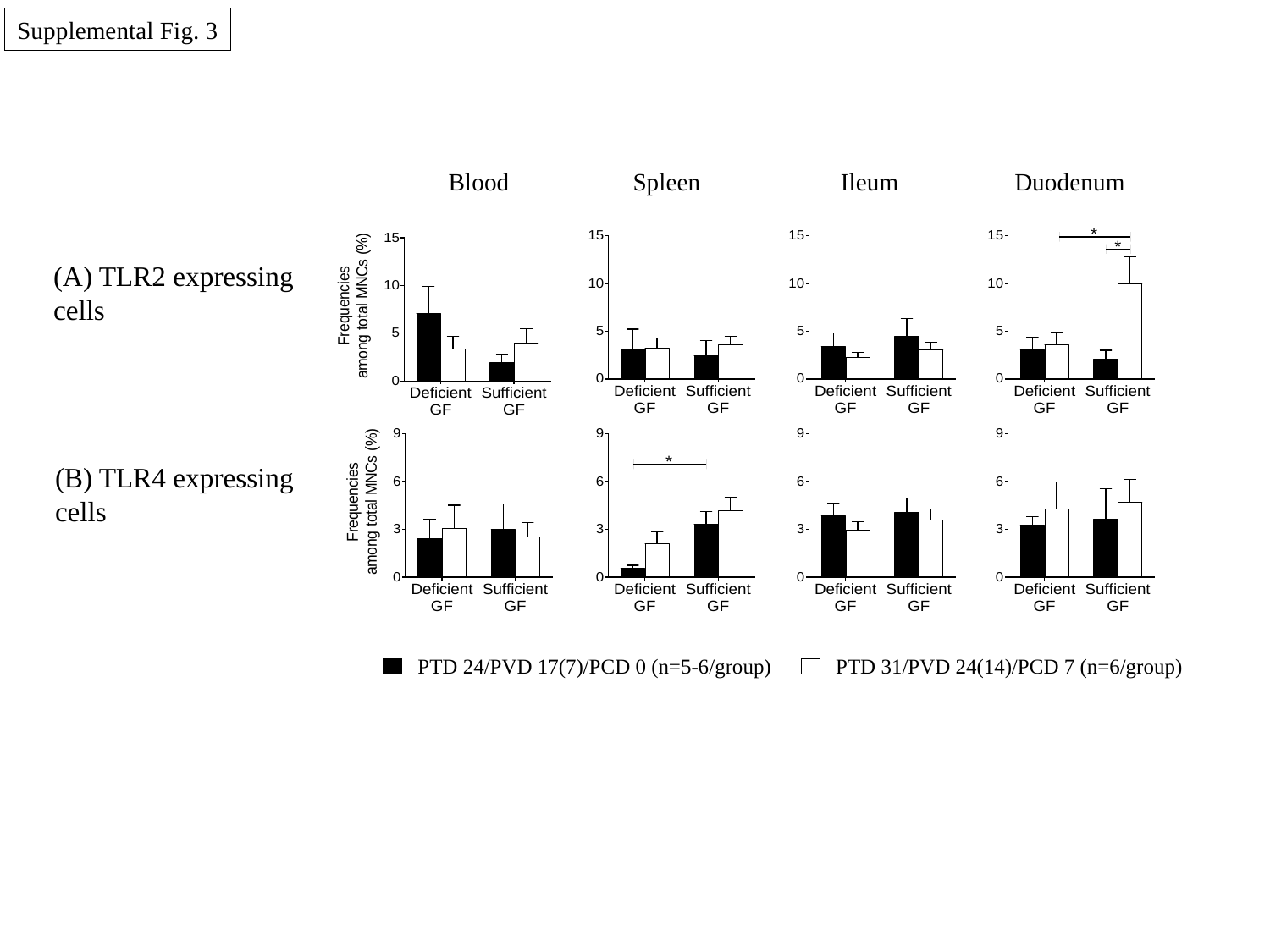

Supplemental Fig. 3
Blood
Spleen
Ileum
Duodenum
(A) TLR2 expressing cells
(B) TLR4 expressing
cells
PTD 24/PVD 17(7)/PCD 0 (n=5-6/group)
PTD 31/PVD 24(14)/PCD 7 (n=6/group)

## Slide 4
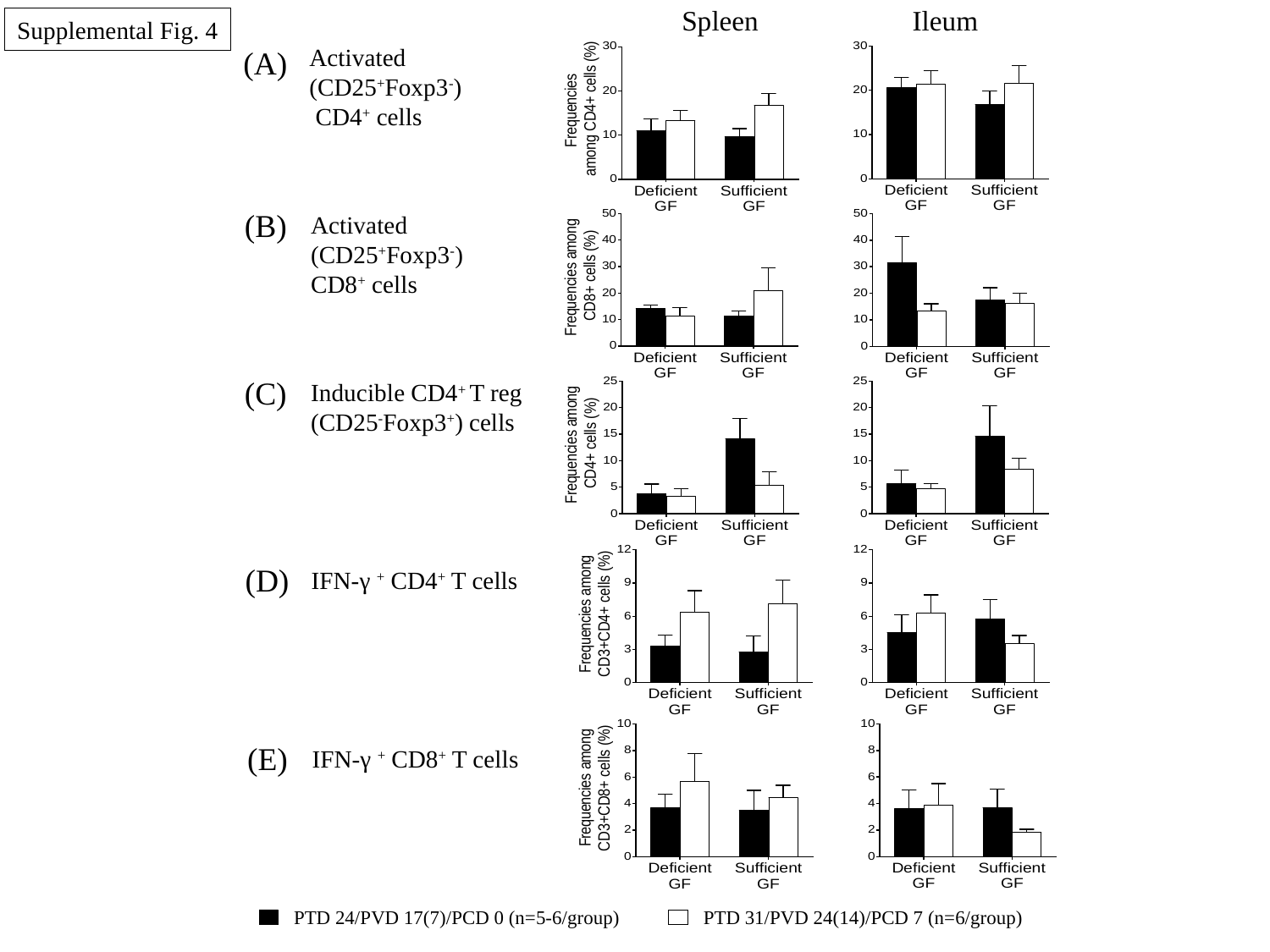

Spleen
Ileum
Supplemental Fig. 4
Activated
(CD25+Foxp3-)
 CD4+ cells
(A)
(B)
Activated
(CD25+Foxp3-)
CD8+ cells
(C)
Inducible CD4+ T reg
(CD25-Foxp3+) cells
(D)
IFN-γ + CD4+ T cells
(E)
IFN-γ + CD8+ T cells
PTD 24/PVD 17(7)/PCD 0 (n=5-6/group)
PTD 31/PVD 24(14)/PCD 7 (n=6/group)

## Slide 5
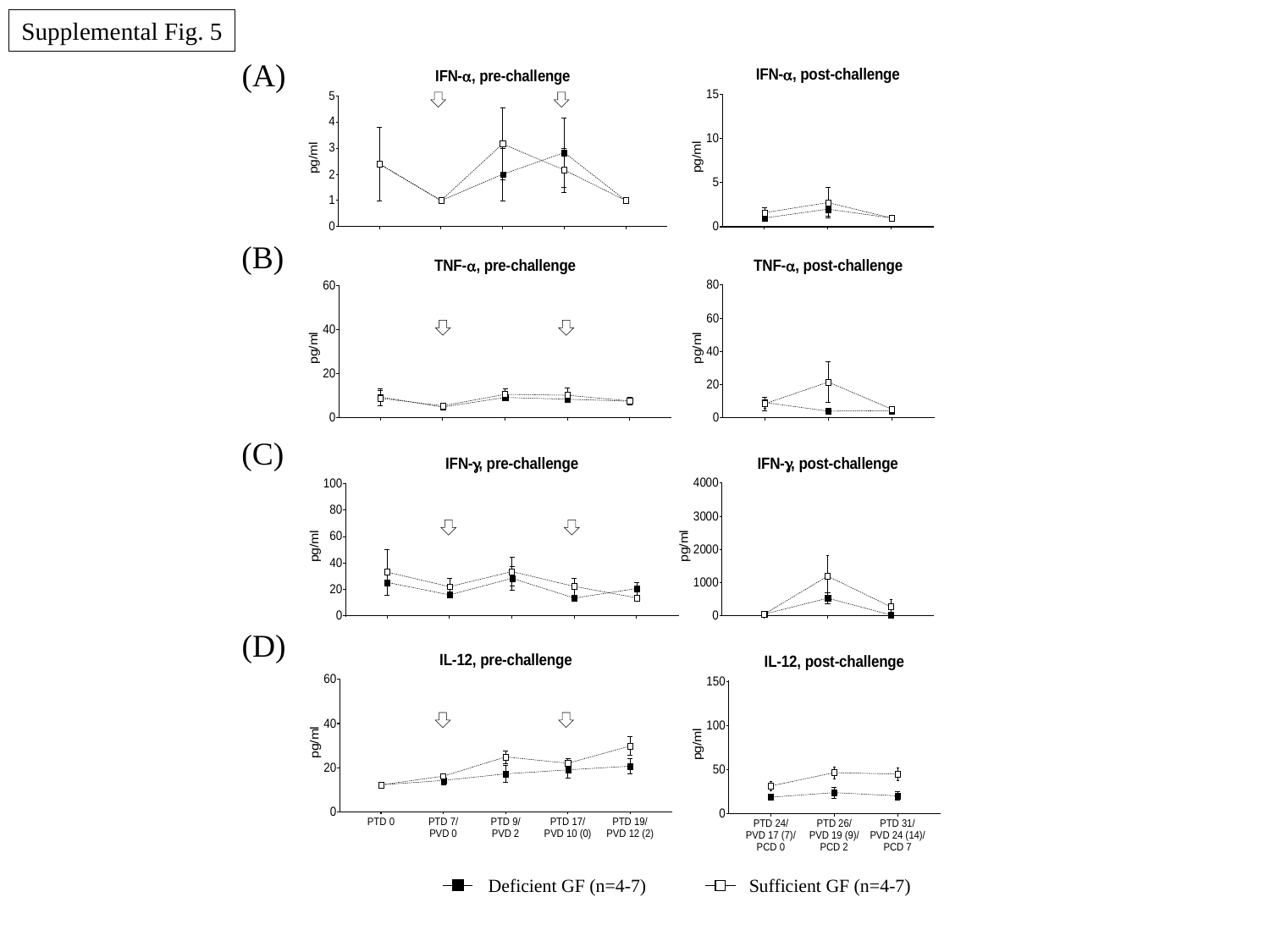

Supplemental Fig. 5
(A)
(B)
(C)
(D)
Deficient GF (n=4-7)
Sufficient GF (n=4-7)

## Slide 6
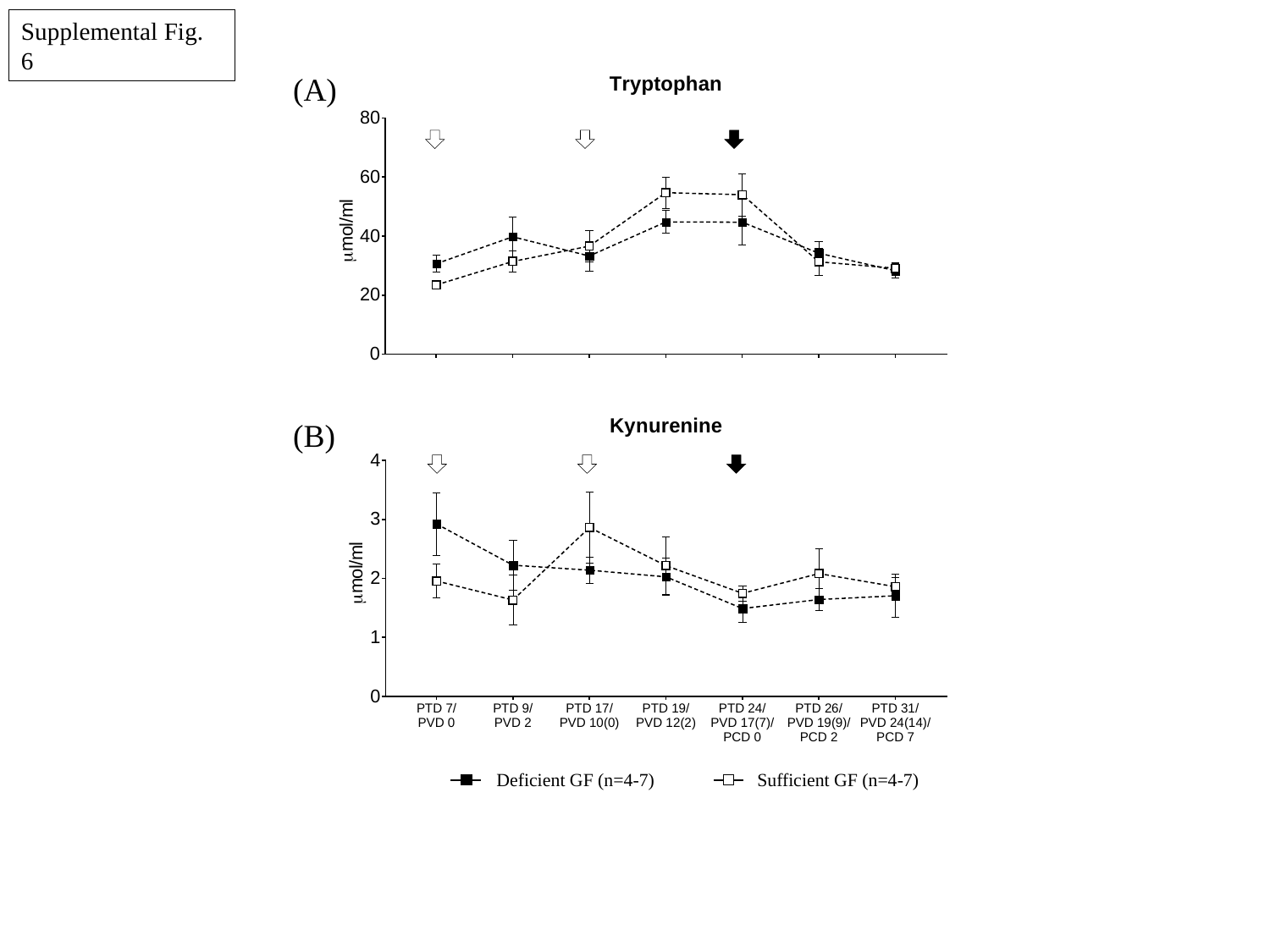

Supplemental Fig. 6
(A)
(B)
Deficient GF (n=4-7)
Sufficient GF (n=4-7)
