# Supplementary material for: Protein deficiency reduces efficacy of oral attenuated human rotavirus vaccine in a human infant fecal microbiota transplanted gnotobiotic pig model
Source: Vaccine. 2018 Oct 8;36(42):6270–81. doi: 10.1016/j.vaccine.2018.09.008 (PMC6180620; doi:10.1016/j.vaccine.2018.09.008)
Supplement: Supplementary data 2 [file mmc2.docx]

**Supplemental Information**

**Supplemental Fig. 1.** Mean frequencies (± SEM) of (A) conventional dendritic cells (cDCs), (B) plasmacytoid dendritic cells (pDCs), and (C) cytotoxic T cells. Significant difference (* *p*<0.05) between germ-free (GF) pigs fed protein deficient diet (Deficient GF) and protein sufficient diet (Sufficient GF) groups or between pre- and post-virulent HRV challenge in each group were determined by Mann-Whitney U test. HRV; human rotavirus, Deficient; protein deficient diet, Sufficient; protein sufficient diet, PTD; post transplantation day, PVD; post 1^st^ (2^nd^) vaccination day and PCD; post challenge day.

**Supplemental Fig. 2.** NK cell function of spleen MNCs in germ-free (GF) pigs fed protein deficient diet (Deficient GF) and protein sufficient diet (Sufficient GF) groups pre- (A) and post-challenge (B) with virulent HRV. Spleen MNCs were co-cultured with carboxyfluorescein succinimidyl ester (CFSE)-stained K562 cells at indicated ratios overnight. The dead cells were stained by incubating with 7-aminoactinomycin D (7-AAD). The cells were observed by flowcytometry to obtain % lysed K562 cells among total K562 cells. Significant differences (* *p*<0.05) between Deficient HIFM and Sufficient HIFM groups were determined by Mann-Whitney U test. HRV; human rotavirus, Deficient; protein deficient diet, Sufficient; protein sufficient diet.

**Supplemental Fig.** **3.** Mean frequencies (± SEM) of MNCs expressing (A) TLR2 and (B) TLR4 in germ-free (GF) pigs fed protein deficient diet (Deficient GF) and protein sufficient diet (Sufficient GF) groups. Mononuclear cells (MNCs) were isolated from blood, spleen, ileum, and duodenum of the piglets from Deficient GF and Sufficient GF groups pre- and post-virulent HRV challenge (PTD 24/PV1D 17/PCD 0 and PTD 31/ PV1D 24/PCD 7, respectively). Significant difference (* p<0.05) between Deficient GF and Sufficient GF groups or between pre- and post-virulent HRV challenge in each group were determined by Mann-Whitney U test. HRV; human rotavirus, Deficient; protein deficient diet, Sufficient; protein sufficient diet, PTD; post transplantation day, PVD; post 1^st^ (2^nd^) vaccination day and PCD; post challenge day.

**Supplemental Fig. 4.** Mean frequencies (± SEM) of activated (CD25^+^Foxp3^-^) cells among CD4^+^ cell (A) and CD8^+^ cell (B) subsets, and inducible T regulatory (CD25^-^Foxp3^+^) cells among CD4^+^ cell subsets (C) and HRV specific IFN-γ producing cells among CD3^+^CD4^+^ (D) and CD3^+^CD8^+^ (E) T cell subsets. Significant difference (* *p*<0.05, ** *p*<0.01) between germ-free (GF) pigs fed protein deficient diet (Deficient GF) and protein sufficient diet (Sufficient GF) groups or between pre- and post-virulent HRV challenge in each group were determined by Mann-Whitney U test. HRV; human rotavirus, Deficient; protein deficient diet, Sufficient; protein sufficient diet, PTD; post transplantation day, PVD; post 1^st^ (2^nd^) vaccination day and PCD; post challenge day.

**Supplemental Fig. 5.** Mean concentrations (± SEM) of innate (IFN-α), proinflammatory (TNF-α), Th1 (IFN-γ and IL-12) and T regulatory (TGF-β) cytokines in serum of germ-free (GF) pigs fed protein deficient diet (Deficient GF) and protein sufficient diet (Sufficient GF) groups before and after virulent human rotavirus challenge. White arrows indicate time points for oral inoculation of attenuated HRV. Significant difference (* *p*<0.05 and ** *p*<0.01) between Deficient GF and Sufficient GF groups at each time point were determined by Mann-Whitney U test. HRV; human rotavirus, Deficient; protein deficient diet, Sufficient; protein sufficient diet, PTD; post transplantation day, PVD; post 1^st^ (2^nd^) vaccination day and PCD; post challenge day.

**Supplemental Fig. 6.** Mean concentrations (± SEM) of tryptophan and kynurenine in serum of germ-free pigs (GF) fed protein deficient diet (Deficient GF) and protein sufficient diet (Sufficient GF) groups. White arrows and a black arrow indicate time points for vaccination by oral attenuated HRV inoculation and virulent HRV challenge, respectively. Significant difference (* *p*<0.05) between Def and Suf groups at each time point were determined by Mann-Whitney U test. HRV; human rotavirus, Deficient; protein deficient diet, Sufficient; protein sufficient diet, PTD; post transplantation day, PVD; post 1^st^ (2^nd^) vaccination day and PCD; post challenge day.
